# Symptomatic Myocarditis Post COVID-19 Vaccination

**DOI:** 10.7759/cureus.24052

**Published:** 2022-04-11

**Authors:** Palak Patel, Dhaval Desai, Nagapratap Ganta, Satish Tadepalli, Priyaranjan Kata, Anish Kanukuntla, Matthew Schoenfeld, Bharath Sathya, Arthur Okere

**Affiliations:** 1 Internal Medicine, Hackensack Meridian Health Ocean University Medical Center, Brick, USA; 2 Cardiovascular Disease, Jersey Shore University Medical Center, Neptune, USA; 3 Internal Medicine, Trinitas Regional Medical Center, Elizabeth, USA; 4 Internal Medicine, Rutgers Health Community Medical Center, Toms River, USA; 5 Interventional Cardiology, Jersey Shore University Medical Center, Neptune, USA; 6 Cardiology, Jersey Shore University Medical Center, Neptune, USA; 7 Cardiology, Hackensack Meridian Health Ocean University Medical Center, Brick, USA

**Keywords:** vaccine adverse event reporting system, vaccine, johnson & johnson, moderna, myocarditis, covid-19

## Abstract

There are few major adverse events after the coronavirus disease 2019 (COVID-19) vaccination. However, increasing cases of myocarditis and pericarditis are being reported to the Vaccine Adverse Event Reporting System (VAERS) in young people, primarily after the second dose of messenger RNA (mRNA) COVID-19 vaccines. We present a case series of myopericarditis post mRNA (Moderna) and myocarditis post vector-based (Johnson & Johnson) COVID-19 vaccines. We intend to highlight the importance of early diagnosis and treatment of vaccine-related myocarditis to reduce mortality and morbidity.

## Introduction

Myocarditis is an inflammation of the heart muscle, while pericarditis is an inflammation of the heart's lining. In all circumstances, the immune system is responding to an infection or another stimulus by inducing inflammation. Chest pain, shortness of breath, and palpitations are all possible symptoms [1]. Postvaccination myocarditis has previously been documented as a rare adverse effect following immunizations, such as smallpox, influenza, hepatitis B, or other vaccines. Myocarditis is diagnosed in about 10 to 20 people per 100,000 in the general population each year, and it affects men more frequently and at a younger age than women [2]. A Centers for Disease Control and Prevention (CDC) Advisory Committee on Immunization Practices recently found a link between the two coronavirus disease 2019 (COVID-19) messenger RNA (mRNA) vaccines from Pfizer-BioNTech and Moderna and myocarditis and pericarditis patients. According to the Advisory Committee on Immunization Practices, there were 1226 reports of suspected myocarditis/pericarditis cases in the Vaccine Adverse Event Reporting System (VAERS) after 300 million COVID-19 mRNA vaccine doses were provided through June 11, 2021, with 67% of those instances occurring after the second dose. Seventy-nine percent of the cases were in men, with the majority occurring in those under the age of 30, with a median age of 24 [2]. Here we are presenting two cases of myocarditis in an 18-year-old male and a 29-year-old male who developed symptoms of myocarditis shortly after their COVID-19 vaccine.

## Case presentation

Patient 1

An 18-year-old male presented with acute onset chest pain and shortness of breath. Chest pain was constant, 7/10, substernal, non-radiating, positional, and pleuritic in nature. The pain was exacerbated by lying down, and alleviated by sitting up, leaning forward, and Tylenol. He had experienced fever, cough, myalgias, dizziness, headaches, nausea, and diarrhea one day prior to presentation. Patient had received his second dose of the mRNA-1273 (Moderna) COVID-19 vaccine a day prior to the onset of symptoms. He denied any adverse events following the first dose of vaccination. On initial presentation, he was afebrile and hemodynamically stable. On physical examination, the patient was alert, oriented to time, place, and person, and not in acute distress. Oral temperature was 98.7 F, blood pressure was 103/67 mm Hg, pulse 84 beats per minute, respiratory rate 18 per minute, and oxygen saturation (SpO2) 98% on room air. Ocular exam was significant for scleral icterus. Cardiac auscultation revealed a normal S1 and S2 with no extra heart sounds. The remainder of the examination was normal. Differential diagnoses were pericarditis, myocarditis, and pleurisy.

**Figure 1:**
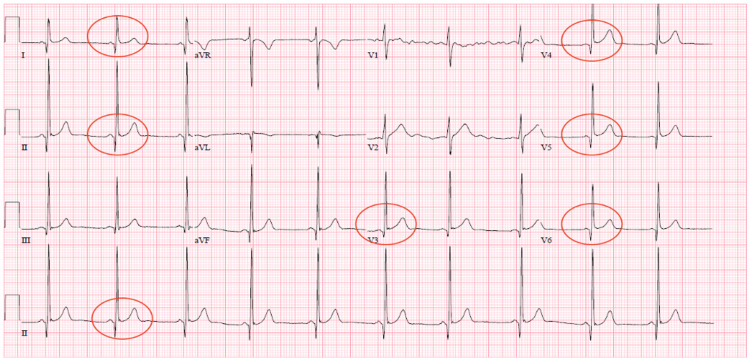
Electrocardiogram of Patient 1 Diffuse ST elevations (circled), with an RSR’ in V1 and V2.

Admission laboratory values are summarized in Table 1. Troponin I was elevated at 1.19 ng/ml. A 12-lead ECG (Figure 1) revealed diffuse ST elevations, with an RSR’ in V1 and V2. COVID-19 test on admission was negative. 

**Table 1:** Laboratory Findings of Patient 1 WBC, white blood cell; BUN, blood urea nitrogen; ALT, alanine aminotransferase; AST, aspartate aminotransferase; ALP, alkaline phosphatase

Laboratory findings	Results	Normal range
WBC	6.7x10^3/uL	4.5-11.0x10^3/uL
Platelet count	140x10^3/uL	140-450x10^3/uL
Hemoglobin	15.1 g/dL	13.2-17.5 g/dL
BUN	12 mg/dL	5-25 mg/dL
Serum creatinine	0.76 mg/dL	0.61-1.24 mg/dL
ALT	29 U/L	10-24 U/L
AST	22 U/L	10-60 U/L
ALP	48 U/L	38-126 U/L
Total bilirubin	4.6 mg/dL	0.2-1.3 mg/dL
Troponin I	1.19 ng/ml.	<0.04 ng/mL

**Figure 2:**
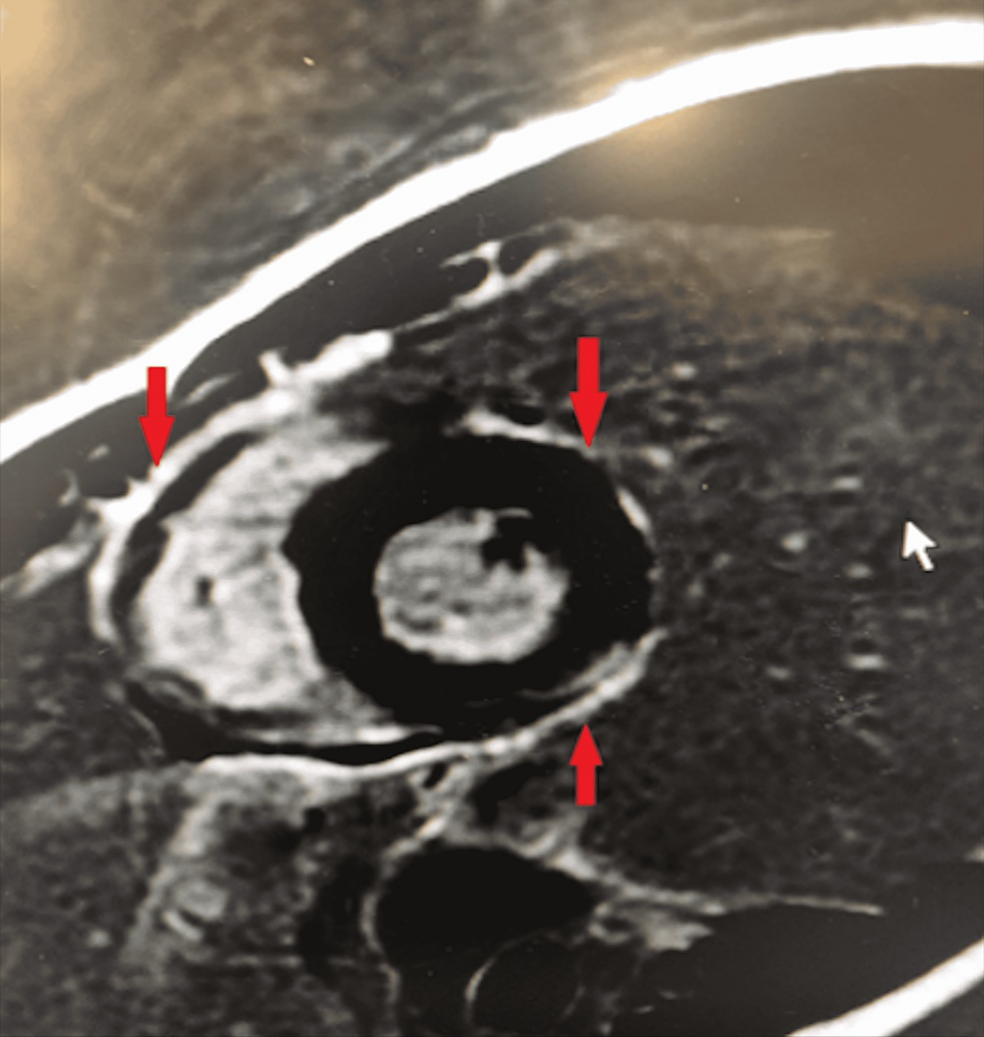
Cardiac Magnetic Resonance Imaging of Patient 1 Sort axis post-contrast image depicting pericardial enhancement (red arrows) adjacent to the right ventricle free wall and the lateral left ventricle wall.

The patient underwent Coronary CTA which revealed normal left ventricle (LV) wall motion and function, normal angiographic coronary arteries, and a calcium score of zero. A 2D echocardiogram was also performed, which revealed normal LV systolic function with an ejection fraction (EF) of 60-65%, without any structural, valvular, or wall motion abnormalities. No ultrasonographic evidence of pericardial or pleural effusion was noted. He also underwent a cardiac MRI (Figure 2), which showed findings consistent with myopericarditis per Lake Louise criteria. Pericardial enhancement adjacent to the right ventricle (RV) free wall and the lateral LV wall was noted. There was a subepicardial pattern of myocardial fibrosis and focal elevations in the myocardial T1 and extracellular volume (ECV) without any definite myocardial edema on T2 mapping. No myocardial infarction was seen on late gadolinium enhancement imaging.

Given the nature of the patient’s presentation, his EKG changes, and relatively quick resolution with intervention, it was deemed that the patient's condition was consistent with myopericarditis, likely secondary to COVID-19 vaccination. He was started on ibuprofen and colchicine for two weeks for the treatment of pericarditis, while supportive care was provided for myocarditis. He was discharged home with instructions to follow up with his cardiologist and primary care physician.

Patient 2

A 29-year-old male presented with acute onset chest pain, shortness of breath, palpitations, and nausea. He reported cleaning his pool and inhaling chlorine gas, immediately after which the patient experienced chest tightness. Chest pain was reported as constant, 8/10, midsternal, pleuritic, positional in nature, pressure-like, with radiation to bilateral arms. Pain was exacerbated by lying down and alleviated by sitting up, leaning forward, and Tylenol. The patient was in good health otherwise and denied any illicit substance use prior to arrival. He reported receiving the Ad26.COV2.S (Johnson & Johnson) COVID-19 vaccine one week prior to initiation of symptoms. The patient denied any adverse events immediately following vaccination.

On physical examination the patient was found to be afebrile and hemodynamically stable on admission. On physical examination, the patient was alert, oriented to time, place, and person, and not in acute distress. Oral temperature was 97.7 degrees Fahrenheit, blood pressure was 131/84 mm Hg, pulse 68 beats per minute, respiratory rate 19 per minute, and SpO2 100% on room air. Cardiac auscultation revealed a normal S1 and S2 with no extra heart sounds. The remainder of the examination was normal. Possible differential diagnoses were pericarditis and non-ST elevation myocardial infarction (NSTEMI).

**Figure 3:**
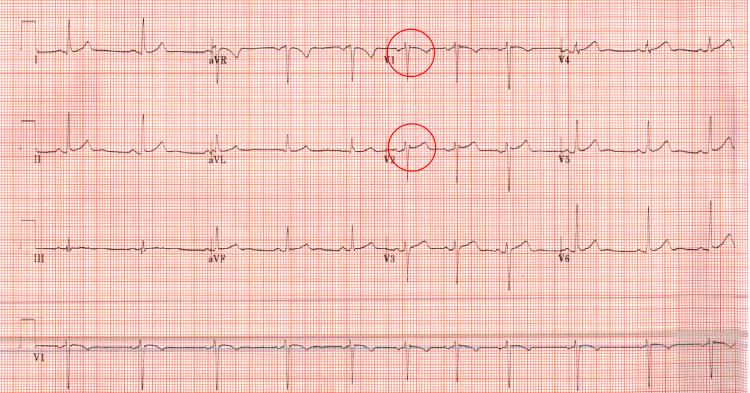
Electrocardiogram of Patient 2 Normal sinus rhythm with an incomplete right bundle branch block (circle).

His laboratory values are summarized in Table 2. Troponin I was elevated at 1.98 ng/ml. A 12-lead EKG (Figure 3) revealed normal sinus rhythm with an incomplete right bundle branch block (RBBB). Due to continued troponin elevation (peaking at 15.65 ng/mL) and persistent recurrent pleuritic, positional chest pain, the patient was started on a heparin drip and transferred to a different facility for an urgent left heart catheterization to rule out ischemia. Cardiac catheterization revealed angiographically normal coronary arteries. He also underwent a transthoracic echocardiogram (TTE), which showed normal LV function with an EF of 60%. No structural, valvular, or wall motion abnormalities were noted. No ultrasonographic evidence of pericardial or pleural effusion was noted. 

**Table 2:** Laboratory Findings of Patient 2 WBC, white blood cell; BUN, blood urea nitrogen; ALT, alanine aminotransferase; AST, aspartate aminotransferase; ALP, alkaline phosphatase; CRP, C-reactive protein; ESR, erythrocyte sedimentation rate

Laboratory findings	Results	Normal range
WBC	4.8x10^3/uL	4.5-11.0x10^3/uL
Platelet count	251x10^3/uL	140-450x10^3/uL
Hemoglobin	14.4 g/dL	13.2-17.5 g/dL
BUN	11 mg/dL	5-25 mg/dL
Serum creatinine	1.12 mg/dL	0.61-1.24 mg/dL
ALT	55 U/L	10-24 U/L
AST	45 U/L	10-60 U/L
ALP	68 U/L	38-126 U/L
Total bilirubin	1.1 mg/dL	0.2-1.3 mg/dL
Troponin I	1.98 ng/mL	<0.04 ng/mL
Creatine kinase	3860 iU/L	22-232 iU/L
CRP	0.33 mg/dL	0-0.74 mg/dL
ESR	10 mm/h	0-15 mm/h
Lipase	25 U/L	20-55 U/L

**Figure 4:**
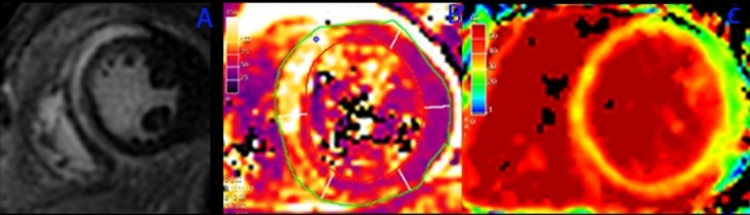
Cardiac Magnetic Resonance Imaging Findings Demonstrative of Myocarditis of Patient 2 Cardiac MRI A-C for patient 2; A) Late gadolinium enhancement imaging (PSIR-LGE) demonstrative of confluent mid-wall and subepicardial myocardial fibrosis in the basal-anterior, basal-septal, and basal-inferior walls. B) T2 parametric map demonstrative of elevated myocardial T2 values in the same distribution as myocardial fibrosis. C) Extracellular volume (ECV) parametric mapping demonstrative of elevated myocardial ECV in the anterior, septal, and inferior walls. The presence of T1 and T2 changes fulfills the Lake Louise criteria to establish a diagnosis of myocarditis.

He also underwent a cardiac MRI (Figure 4) which showed findings consistent with myocarditis per Lake Louise criteria. A subepicardial pattern of myocardial fibrosis was seen on late gadolinium enhancement imaging in multiple wall segments, involving 26% of the myocardial mass. There were numerous wall segments with elevated myocardial T2 values on parametric mapping. There were markedly elevated myocardial ECV values. A small pericardial effusion was also seen. 

Once the patient’s pain subsided and troponin trended down, he was discharged home in stable condition with colchicine, ibuprofen, and famotidine for three months with instructions to follow up with his cardiologist and primary care physician.

## Discussion

The novel beta-coronavirus, severe acute respiratory syndrome coronavirus 2 (SARS-CoV-2), which emerged in 2019 in Wuhan, China, has caused human-to-human transmission at an unprecedented rate. Beta-coronaviruses have jumped between the species and caused three zoonotic outbreaks, namely SARS-associated coronavirus (SARS-CoV) (2002-2003), Middle East respiratory syndrome (MERS)-CoV (2012), and SARS-CoV-2 (2019 till date) in the last two decades. Myocarditis is common during viral infection, with cases described as early as the influenza pandemic of 1917, and the COVID-19 pandemic is no exception. Myocarditis is one of the known manifestations of COVID-19 and was even reported during the SARS and MERS outbreaks.

Myocarditis is an inflammation of the middle layer of the heart that causes myocardial damage without ischemia [3]. The immune system mistaking the vaccine's mRNA for an antigen, initiating pro-inflammatory cascades and immunological pathways in the heart, is thought to be the pathophysiology of vaccine-related myocarditis. Although nucleoside changes reduce mRNA's innate immunogenicity, the immunological response to mRNA may still trigger an aberrant innate and acquired immune response, which could explain why mRNA vaccines elicit a stronger immune response than other COVID-19 vaccines. Antibodies against SARS-CoV-2 spike glycoproteins may react with structurally comparable human protein sequences, such as cardiac-myosin heavy chains [4]. The proposed mechanism includes direct injury to myocytes by the virus, the cytotoxic T lymphocyte-related immune response, and cytokine storm. Myopericarditis after vaccination has been previously reported sporadically in the medical literature: after Haemophilus influenza type b and hepatitis B vaccination among persons aged < 19 years; after smallpox vaccination among persons aged 19-49 years; and after inactivated influenza and live attenuated zoster vaccines among persons aged ≥ 50 years [5]. Despite the introduction of new vaccines over the years, myopericarditis remains rarely reported after licensed vaccination in the United States. Overall, incidences after smallpox and anthrax vaccines were most commonly reported. 2D echocardiogram and Cardiac MRI are diagnostic modalities for myocarditis in patients with COVID-19. Myocardial edema, necrosis, and late gadolinium enhancement are pathognomonic. 

Our case series describes cases of myopericarditis and myocarditis after immunization (as illustrated in Table 3), with vaccines with completely different mechanisms leading to similar clinical presentation.

**Table 3:** Summary of Diagnostics for Myocarditis Post COVID-19 Vaccination mRNA; messenger RNA, LV; left ventricle, RBBB; right bundle branch block, EKG; electrocardiogram, MRI; magnetic resonance imaging

	Patient 1	Patient 2
Vaccine received	mRNA-1273 (Moderna)	Ad26.COV2.S (Johnson & Johnson)
Highest troponin	1.19	15.65
EKG changes	Diffuse ST elevations, RSR’ in V1 and V2	Normal sinus rhythm with incomplete RBBB
Cardiac MRI findings	1) Subepicardial pattern of myocardial fibrosis and focal elevations in the myocardial T1 and (Extra Cellular Volume) 2) Enhancement of the pericardium adjacent to the RV free wall and adjacent to the lateral LV wall 3) Small pericardial effusion	1) Subepicardial pattern of myocardial fibrosis on late gadolinium enhancement imaging in multiple wall segments, involving 26% of the myocardial mass 2)Numerous wall segments with elevated myocardial T2 values on parametric mapping 3)Markedly elevated myocardial ECV (Extra Cellular Volume) values 4)Small pericardial effusion

The rapid development of immunization against COVID-19 using newer technology marks a new milestone in medical history. Of the multiple epitopes, the spike (S) glycoprotein is a commonly selected target for COVID-19 vaccine development [6], since it is the major SARS-CoV-2 surface protein and mediates viral entry by binding to the angiotensin-converting enzyme 2 (ACE2) receptor on host cells. 

The mRNA-1273 (Moderna) and BNT162b2 (Pfizer) vaccines use modified mRNA protected by lipid nanoparticles. These are injected through an intramuscular route, leading to uptake by host cells, followed by expression of the mRNA producing spike protein against which host immune response is developed. Ad26.COV2.S (Johnson & Johnson) COVID-19 vaccine is a vector-based vaccine using disabled adenovirus-26 to transmit dsDNA, which is translated into mRNA in the host cell's nucleus to produce viral spike proteins against which the host mounts antibody response. Table 4 summarizes the advantages and limitations of both types of vaccines [7]. Theoretically, both of these vaccines lead to the production of viral spike protein against which the host mounts an antibody response, leading to the development of immunity. In the clinical trials, adverse events were reported more frequently one to two days after the second dose. 

**Table 4:** Advantages and Limitations of Different Types of Vaccines MERS-CoV: Middle East respiratory syndrome coronavirus

	mRNA vaccines [mRNA-1273 (Moderna) & BNT162b2 (Pfizer)]	Vector-based vaccines [Ad26.COV2.S (Johnson and Johnson)]
Advantages	1) Translation of mRNA occurs in the cytosol of the host cell averting the risk of any sort of integration into the host genome	1) Avoids handling of any infectious particle and it has been used widely for MERS-CoV with positive results from the trials 2) Show a highly specific gene delivery into the host cell with a vigorous immune response
Limitations	1) Safety issues with reactogenicity have been reported for various RNA based vaccines 2) It also shows instability	1) The host may possess immunity against the vector due to prior exposure, reducing the efficacy. 2) May lead to cancer due to the integration of the viral genome into the host genome

Our report on the occurrence of myocarditis after COVID-19 vaccines with different action mechanisms can be considered an adverse reaction following immunization. This reaction may be related to the COVID-19 spike protein expressed after vaccination rather than the technology utilized for manufacturing the vaccine. Accordingly, it will be critical to collect more data as vaccine use becomes more widespread amongst young individuals. Although the disease course in our patient was mild, myocarditis can have a much more severe presentation with possible future sequelae. Ideal timing and screening strategies for these patients will need to be determined. 

## Conclusions

Our report on the occurrence of myocarditis after COVID-19 vaccines with different mechanisms of action suggests reaction may be related to the COVID-19 spike protein expressed and host antibody response to the spike protein. As vaccines for COVID-19 become more widely distributed, it will be essential to collect more data on the topic. Currently, the greater public health benefits of vaccination outweigh the risk of rare occurrences described herein.
